# Inhibition of the Mitochondrial Carnitine/Acylcarnitine Carrier by Itaconate through Irreversible Binding to Cysteine 136: Possible Pathophysiological Implications

**DOI:** 10.3390/biom13060993

**Published:** 2023-06-15

**Authors:** Nicola Giangregorio, Annamaria Tonazzi, Lara Console, Mariafrancesca Scalise, Cesare Indiveri

**Affiliations:** 1 National Research Council (CNR), Institute of Biomembranes, Bioenergetics and Molecular Biotechnologies (IBIOM), Via Amendola 122/O, 70126 Bari, Italy; a.tonazzi@ibiom.cnr.it; 2 Unit of Biochemistry and Molecular Biotechnology, Department DiBEST (Biologia, Ecologia, Scienze della Terra), University of Calabria, Via Bucci 4C, 87036 Arcavacata di Rende, Italy; lara.console@unical.it (L.C.); mariafrancesca.scalise@unical.it (M.S.)

**Keywords:** mitochondria, SLC25A20, carnitine, carrier, itaconate, cysteines, ROS

## Abstract

Background: The carnitine/acylcarnitine carrier (CAC) represents the route of delivering acyl moieties to the mitochondrial matrix for accomplishing the fatty acid β-oxidation. The CAC has a couple of Cys residues (C136 and C155) most reactive toward ROS and redox signaling compounds such as GSH, NO, and H_2_S. Among physiological compounds reacting with Cys, itaconate is produced during inflammation and represents the connection between oxidative metabolism and immune responses. The possible interaction between the CAC and itaconate has been investigated. Methods: the modulatory effects of itaconate on the transport activity of the native and recombinant CAC were tested using the proteoliposome experimental model together with site-directed mutagenesis and computational analysis. Results: Itaconate reacts with the CAC causing irreversible inhibition. Dose–response experiment performed with the native and recombinant protein showed IC_50_ for itaconate of 11 ± 4.6 mM and 8.4 ± 2.9 mM, respectively. The IC_50_ decreased to 3.8 ± 1.0 mM by lowering the pH from pH 7.0 to pH 6.5. Inhibition kinetics revealed a non-competitive type of inhibition. C136 is the main target of itaconate, as demonstrated by the increased IC_50_ of mutants in which this Cys was substituted by Val. The central role of C136 was confirmed by covalent docking. Administration of dimethyl itaconate to HeLa cells inhibited the CAC transport activity, suggesting that itaconate could react with the CAC also in intact cells.

## 1. Introduction

Oxidative stress, implicated in biological processes ranging from immune function to aging, is often accompanied by excess mitochondrial ROS (mROS) production leading to the oxidation of enzymes and transmembrane proteins with consequent impairment of metabolic pathways. Some enzymes and membrane proteins are particularly sensitive to ROS due to specific structural features that make these proteins more reactive toward free radicals and/or oxidative compounds. An increase in ROS is followed by the production of molecules that signal the altered redox state and regulates the activity of specific proteins for reversing and/or buffering the increase in ROS [1,2,3].

Among mitochondrial proteins, the carnitine/acylcarnitine carrier (CAC, SLC25A20) that shuttles acyl moieties in the matrix, via a ping-pong mechanism [4], to accomplish the fatty acid β-oxidation pathway [5], represents an excellent sensor of redox signaling molecules. Several physiological compounds modulate the CAC transport function by targeting Cys residues. Old studies on intact mitochondria already showed the high reactivity of the CAC toward alkylating chemical reagents such as N-ethylmaleimide [6]. Then, the reactivity of the transporter toward alkylating and mercury-related compounds was characterized in a proteoliposome experimental model harboring the transporter purified from rat liver mitochondria [7]. In the last ten years, by using integrated approaches of intact mitochondria, cell lines, site-directed mutagenesis, and bioinformatics, the CAC has been shown to sense thiol-reactive physiological agents such as hydrogen peroxide, nitric oxide, hydrogen sulfide, glutathione and, very recently, carbon monoxide [8,9,10,11,12]. The response to these physiological effectors is mediated by a crucial couple of Cys resides, i.e., C136 and C155, located in a peculiar microenvironment that makes these two residues highly sensitive to thiol-reactive compounds giving the CAC a specific relevance to oxidative and/or inflammatory processes [9,10,11].

Among physiological compounds signaling altered metabolic conditions and reacting with Cys residues, itaconate is produced during inflammation. It is formed following the decarboxylation of cis-aconitate, an intermediate of the Tricarboxylic Acid Cycle through the mitochondrial enzyme Immune-Responsive Gene 1 (Irg1), the overexpression of which is induced in macrophages during inflammatory states. Production of itaconate connects cell metabolism to oxidative and immune responses [13,14]. Itaconate contains an electrophilic unsaturated α-β carboxylic acid that alkylates cysteine residues of proteins [15]. It covalently binds cysteine residues of the KEAP1 protein, allowing Nrf2 to increase the expression of downstream genes with antioxidant and anti-inflammatory properties [16]. Cell culture studies also suggest that itaconate regulates inflammation through its inhibitory effects on the production of cytokines and ROS [17]. One of the mechanisms by which itaconate prevents ROS production is based on the inhibition of succinate dehydrogenase (SDH) [18,19]. Itaconate also reacts with GSH, leading to a decrease in concentration and then giving the opposite effect of increasing ROS concentration [19].

Interestingly, it was shown that itaconate, which is formed in the mitochondrial matrix, can be transported by three mitochondrial carriers, i.e., the citrate (SLC25A1), the oxoglutarate (SLC25A11), and the dicarboxylate carriers (SLC25A10) [15], thus being available in the intermembrane space and the cytosol. This correlates well with the effect of itaconate and its derivatives on the glycolysis enzymes [20,21]. Owing to the described sensitivity of the CAC to thiol-reacting compounds, in particular alkylating compounds, and to the possible role of this transporter in mediating a response of the fatty acid β-oxidation to the cell redox state, we investigated the reactivity of itaconate toward the CAC, performing in vitro and ex vivo experiments.

## 2. Materials and Methods

### 2.1. Materials

L-[methyl-^3^H]carnitine and L-[2,3-^3^H]ornithine from Scopus Research BV Costerweg, Sephadex G-75, egg-yolk phospholipids (l-α phosphatidylcholine from fresh turkey egg yolk), PIPES, HEPES, Triton X-100, cardiolipin, L-carnitine, L-ornithine, sodium itaconate, dimethyl itaconate (DMI), N-ethylmaleimide (NEM) were purchased from Sigma-Aldrich, Milan, Italy. All other reagents were of analytical grade.

### 2.2. Overexpression of the WT and Mutant CACs

As previously described, the pMW7-WTratCAC recombinant plasmid was used for over-expressing the rat CAC [22]. The overlap extension method was used to introduce the mutations; in the main time, NdeI and HindIII restriction sites were also added using appropriate primers [23]. The PCR products were purified by the QIAEX II Gel Extraction Kit (QIAGEN) and digested with NdeI and HindIII and then ligated into the pMW7. All mutations were verified by DNA sequencing. The resulting plasmid constructs were transformed into *E. coli* C0214. Overexpression, inclusion body fraction preparation, and purification of the CAC proteins were performed as previously described [24].

### 2.3. Cloning, Overexpression, and Isolation of hORC1Wild Type Protein

The 903 bp cDNA coding for the hORC1Wild Type (WT) protein was amplified from the Mega Man Human Transcriptome Library with the forward and reverse primers 5′-GGAATTCCATATGAAATCCAATCCTGCTATCCAG-3′ and 5′-CCCAAGCTTGTATGCTTCCAACTGGTTCATC-3′, containing the NdeI and HindIII sites, respectively. The amplified cDNA was then cloned in the NdeI/HindIII sites of the pET-21a(+) expression vector. The resulting recombinant plasmid, defined as pET-21a(+)-hORC1, encodes a full-length hORC1 protein with a His6 tag before the termination codon. Bacterial overexpression of WT was performed, in *E. coli* C0214, as described above for the carnitine/acylcarnitine carrier [24]. hORC1 Wild Type protein, as inclusion body fraction, was solubilized and then purified by Ni-chelating chromatography (NiNTA Resin) as described previously [25].

### 2.4. Reconstitution of the CAC, ORC1, and ANT in Proteoliposomes

As previously described, recombinant CAC (WT and mutants) and ORC1 proteins were used for proteoliposome formation [24]. For experiments with native CAC, ORC1, or ANT proteins, rat liver mitochondria, or HeLa cell mitochondria, the latter for the CAC alone, were first purified using the standard procedure of cell disruption and differential centrifugation [26]. Then proteins were extracted using 3% Triton X-100 and reconstituted as described for the recombinant proteins. The concentration of intraliposomal carnitine and ornithine was 15 mM and 30 mM, respectively, in all samples.

### 2.5. Transport Assay in Proteoliposomes or Intact Mitochondria

To test the transport activity of the CAC, 550 μL of proteoliposomes was passed through a Sephadex G-75 column to remove the external substrate. Six hundred μL of the turbid eluate was collected, divided into samples of 100 μL each, and used for transport measurement by the inhibitor-stop method [27]. Transport was started by adding 0.1 mM [^3^H]carnitine ([^3^H]ornitine or [^3^H]ATP) to proteoliposome samples and stopped with 1.5 mM NEM (N-ethylmaleimide) at the indicated time interval. In the control samples, NEM was added with the labeled substrate at time zero. Transport rates were measured in 10 min, i.e., within the initial linear range of the time course, at 25 °C. After terminating the transport reaction, the external substrate was removed by chromatography on the Sephadex G-75 column, and the intraliposomal radioactivity was measured [27]. The experimental values were corrected by subtracting the controls.

### 2.6. HeLa Cells Treatment with DMI

HeLa cells were maintained in Dulbecco’s Modified Eagle Medium (DMEM) supplemented with 10% (*v*/*v*) fetal bovine serum (FBS), 1 mM glutamine, and 1 mM sodium pyruvate and Pen/Strep as antibiotics. Cells were grown on 10 cm^2^ plates at 37 °C in a humidified incubator and CO_2_. Cells were treated with 10 mM dimethyl itaconate (DMI) or vehicle for 45 min. After extensive washing, cells were harvested, and mitochondria were prepared with a fractionation procedure [28].

### 2.7. Covalent Docking

The three-dimensional coordinates of the CAC homology model were refined and prepared within Maestro 2020_2 using Schrödinger Protein Preparation tool. Default parameters were used. Itaconate was downloaded from PubChem in sdf format and subsequently prepared using LigPrep by optimizing geometries and assigning appropriate protonation states. Epik with default parameters was used [29,30]. CovDock workflow was performed [31] using a receptor grid generated keeping the default parameters. The cubic box was centered around C136 and K135. The Michael Addition was selected as the reaction mode. Docking results visualization was performed with the UCSF Chimera v.1.14 software (Resource for Biocomputing, Visualization, and Informatics, University of California, San Francisco, CA, USA) [32].

### 2.8. Other Methods

The amount of protein was estimated by using the Lowry protein assay, modified for the presence of non-ionic detergents [33].

### 2.9. Statistical Analysis

Statistical analysis was performed by Student’s *t*-test, as indicated in figure legends. Values of * *p* < 0.05 and ** *p* < 0.01 were considered statistically significant. Data points were derived from three different experiments as specified in the figure legends.

## 3. Results

### 3.1. Effect of Itaconate on the Native or the Recombinant CAC

**Figure 1:**
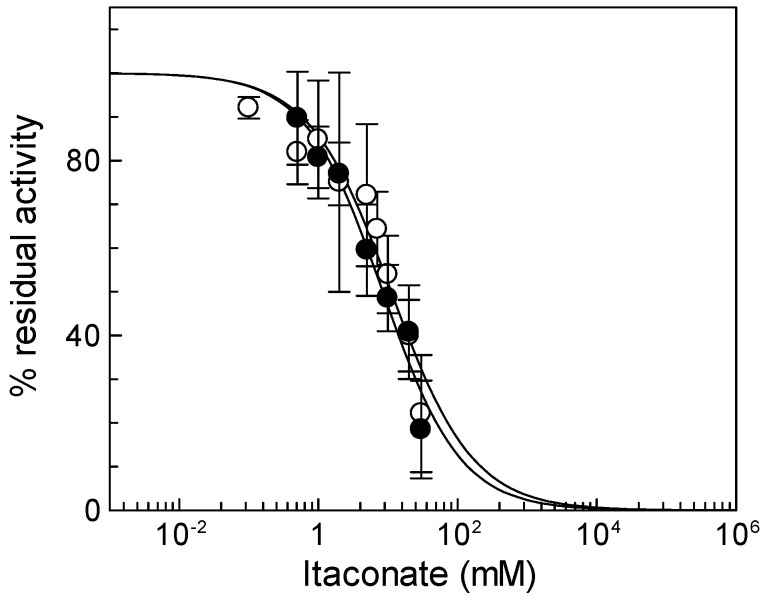
Effect of itaconate on the native and recombinant mitochondrial carnitine/acylcarnitine (CAC). Dose–response analysis for itaconate inhibition was carried out using proteoliposomes reconstituted with native (○) or recombinant (●) CAC carriers. After 10 min of itaconate incubation, transport activity was started by adding 0.1 mM [^3^H]carnitine and stopped after 10 min as described in Section 2. Percent of residual activity with respect to the control, without itaconate treatment, was reported. The values are the means ± SD from three independent experiments.

To check the reactivity of the native mitochondrial carnitine/acylcarnitine carrier (CAC) toward itaconate, rat liver mitochondria were solubilized with Triton X-100, and the mitochondrial protein extract was reconstituted in proteoliposomes for assaying the CAC transport activity in the presence of itaconate (see Materials and Methods). Figure 1 shows the dose–response experiment of ^3^[H]carnitine/carnitine antiport, mediated by the native CAC, to itaconate, from which an IC_50_ of 11 ± 4.6 mM was derived. The dose–response experiment was also performed on the recombinant CAC (Figure 1), from which an IC_50_ of 8.4 ± 2.9 mM was obtained. The IC_50_ values of the native and the recombinant CAC are very similar, in agreement with previous data indicating that the functional properties of the recombinant CAC are coincident with those of the native protein [34]. Thus, the recombinant CAC was used for the other experiments of the present work.

### 3.2. pH Dependence of Itaconate Effect

**Figure 2:**
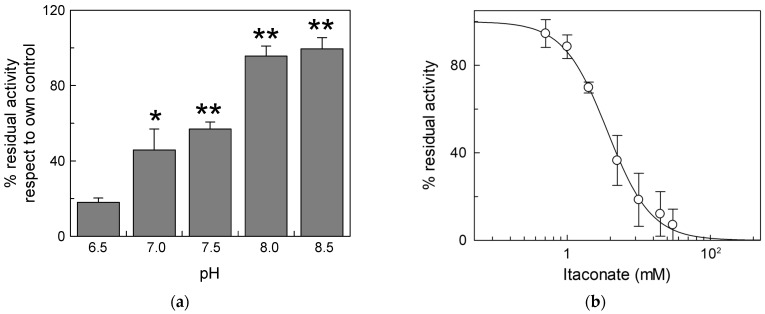
Effect of different pH on the inhibition of CAC protein by itaconate. (**a**) Proteoliposomes reconstituted with the recombinant CAC were incubated at various pHs (range pH 6.5–8.5) for 10 min with 10 mM itaconate. (**b**) Dose–response analysis of inhibition by itaconate of the recombinant CAC at pH 6.5. was performed. Proteoliposomes were incubated for 10 min at pH 6.5 with itaconate at the indicated concentrations. In both (**a**,**b**), the transport activity was measured for 10 min by adding to proteoliposomes 0.1 mM [^3^H]carnitine. The data are expressed as a percent of the controls (no addition of itaconate) and are means ± SD from three independent experiments, significantly different from the respective control as estimated by Student’s *t*-test (* *p* < 0.05, ** *p* < 0.01).

Due to the activity of the respiratory complexes, the pH of the intermembrane space is more acidic than that of the matrix, thus exposing the external side of mitochondrial carriers and, hence, of the CAC, to acidic pH [35]. For this reason, we carried out experiments of inhibition by itaconate at different pH (Figure 2). The effect of itaconate was more pronounced (about 80% inhibition) in proteoliposomes incubated at pH 6.5. The efficiency of itaconate in inhibiting the carrier decreased drastically at a more alkaline pH. Virtually no inhibition was observed at pH 8.0 and pH 8.5. Probably, the local pH influences the pKa of the Cys residues or the microenvironment surrounding reactive Cys, thus favoring the reaction with itaconate [36]. The dose–response experiment of the WT protein was then performed at pH 6.5 (Figure 2b). The calculated IC_50_ value, 3.8 ± 1.0 mM, is lower than that derived at pH 7.0.

### 3.3. Identification of the Residue Responsible for Itaconate Action

**Figure 3:**
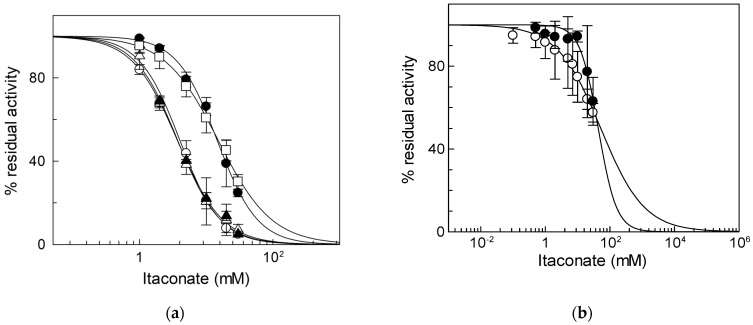
Identification of residue responsible for itaconate inhibition. (**a**) Dose–response analysis of the Cys mutants of CAC for the inhibition by itaconate was performed. Transport activity of the WT (○), C136A (●), C155A (Δ), C23V/C58V/C89S/C155V/C283S (▲), and C23V/C58V/C89S/C136V/C155V/C283S (□) was measured by adding 0.1 mM [^3^H]carnitine after treatment by itaconate at the indicated concentrations. Transport activity (µmol/10 min/mg) is: WT 1.80 ± 0.31; C136A 0.91 ± 0.10; C155A 1.00 ± 0.17; C23V/C58V/C89S/C155V/C283S 1.02 ± 0.159; C23V/C58V/C89S/C136V/C155V/C283S 0.71 ± 0.22. (**b**) Dose–response analysis of proteoliposomes reconstituted native (○) or recombinant ORC (●) incubated with itaconate at the indicated concentrations. Transport activity was measured by adding 0.1 [^3^H]ornithine, respectively, as described in Materials and Methods. The data are expressed as a percent of the controls (no addition of itaconate) and are means ± SD from three independent experiments.

To identify the critical cysteine residues of the CAC involved in the binding with itaconate, WT and Cys mutants were tested, harboring or lacking specific Cys residues already known to be redox sensors of the CAC [34,37]. In particular, we hypothesized that the major target of itaconate could be C136 since this Cys residue is the site of interaction of N-ethylmaleimide [38], which is an alkylating reagent, as well. To prove the involvement of this Cys residue, mutants containing more than one Cys substitution had to be used. However, the substitution of more than four Cys residues with Ser led to inactive proteins. Thus, to overcome this problem, some of the Cys residues were substituted with Val [8]. Thus, we initially tested the three proteins containing C136, i.e., the WT and the mutants C155A and C23V/C58V/C89S/C155V/C283S (containing the sole C136). The IC_50_ values derived from the dose–response curves of the two mutants, 3.8 ± 0.64 mM for the C155A mutant or 4.0 ± 0.14 mM for the C23V/C58V/C89S/C155V/C283S (Figure 3a), were very similar to that of WT. On the contrary, the IC_50_ of the mutant lacking C136 (C136A) was about four times (15 ± 2.1 mM) that of C136 containing proteins and equal to that of the C23V/C58V/C89S/C136V/C155V/C283S (C-less) mutant that is 15 ± 2.4 mM (Figure 3a). This datum demonstrates that C136 is the specific target for itaconate.

To verify if the observed inhibition is a peculiarity of the CAC, the same experiment was performed on another mitochondrial carrier, the ornithine/citrulline carrier (ORC, SLC25A15). ORC was chosen since it shares 36.5% identity with the CAC and is one of the mitochondrial carriers with the greatest number of cysteine residues (9 Cys) [39], one of which, i.e., C132, is located in a position corresponding to that of C136 in the CAC [10]. The IC_50_ value of native ORC for itaconate is 52 ± 5.5 mM (Figure 3b), indicating that ORC has a much lower sensitivity for inhibition by itaconate than the CAC. The large difference in IC_50_ between the CAC and ORC was also observed on the recombinant proteins; indeed, an IC_50_ value of 44 ± 6.0 mM was derived for the recombinant ORC (Figure 3b). Moreover, the effect of itaconate was also tested on another mitochondrial carrier belonging to the same SLC25 family, the adenine nucleotide translocase (ANT). This carrier was chosen since, like the CAC, possess cysteine residues that can react with alkylating reagents such as ethylmaleimide [40]. The IC_50_ value of native ANT for itaconate was 204 ± 95 mM (Figure S1). Since all other mitochondrial carriers do not harbor Cys residues in positions corresponding to those of the C136 of the CAC [10], these data suggest the specificity of itaconate action on the CAC.

From Figure 3a, it appears that inhibition by itaconate, even though at a much lower extent, was observed even in the absence of Cys residues, i.e., in the C-less protein, indicating that a low affinity interaction of the CAC with itaconate may occur with other amino acid residues.

**Figure 4:**
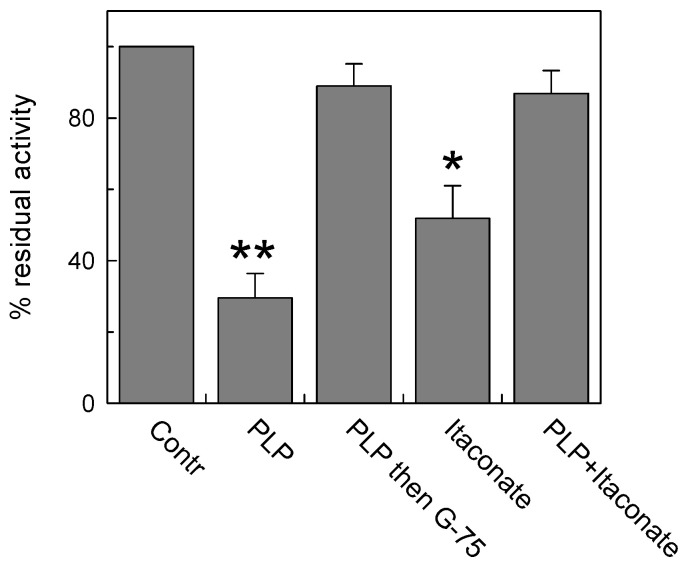
Lysine contribution to CAC inhibition by itaconate. Protection of PLP (pyridoxal phosphate) from inhibition by itaconate on C-less CAC was carried out. C-less mutant (i.e., C23V/C58V/C89S/C136V/C155V/C283S) was reconstituted into proteoliposomes and treated or not treated with 30 mM PLP for 10 min (“Contr” or “PLP” column, respectively). The third column (“PLP then G-75”) showed the effect of PLP removal by size exclusion chromatography on the residual transport activity. The column “Itaconate” represents the effect of 15 mM itaconate on proteoliposomes not preincubated with PLP. The last column represents the effect of the addition of 15 mM itaconate on PLP pretreated proteoliposomes. A second size exclusion chromatography was performed prior to measure the transport activity. The activity assay was performed by adding to proteoliposomes 0.1 mM [^3^H]carnitine and stopped after 10 min. The data are means ± SD from three independent experiments. Student’s *t*-test (* *p* < 0.05, ** *p* < 0.01).

In this respect, Lys is a good candidate owing to its ability to undergo an alkylation reaction [41]. To address this point, we performed a pretreatment with pyridoxal 5′-phosphate (PLP), which is known to bind Lys reversibly (and Arg), to protect these residues from interaction with itaconate. Indeed, PLP forms a reversible aldimine (Shiff base) with the amino-terminal group of Lys residues that can be reversed by removing the reagent from proteoliposomes by Sephadex size exclusion chromatography (G-75). Treatment with 30 mM PLP for 10 min inhibited the C-less CAC protein by about 75% of the control, in agreement with previous data on the native protein [42] (Figure 4). Then, removal of PLP by size exclusion chromatography fully reversed the transport function in agreement with the reversible nature of the aldimine bond. The addition of 15 mM itaconate also inhibited the C-less CAC, in agreement with the data of Figure 3, but the addition of itaconate to the PLP pretreated protein and the subsequent removal of the reactants by G-75 unveiled the inefficacy of itaconate in inhibiting the “protected” transporter (Figure 4). This demonstrates that itaconate reacts, with low affinity, with the Lys residue(s) protected by PLP.

### 3.4. Irreversible Nature of Itaconate Interaction with the CAC

**Figure 5:**
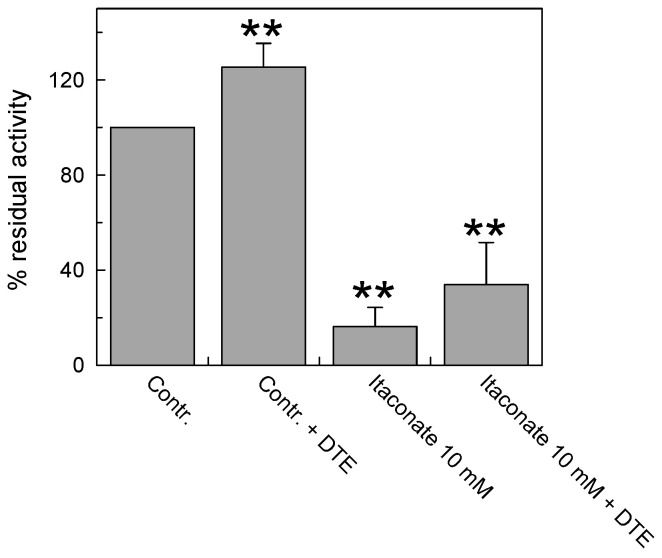
Effect of the reducing agent DTE on the inhibition of recombinant CAC protein by itaconate. The carnitine homo-exchange transport activity was measured, as described in Materials and Methods, adding 0.1 mM [^3^H]carnitine to proteoliposomes reconstituted with the recombinant WT CAC containing 15 mM internal carnitine in the absence (“Contr”) or the presence of 10 mM itaconate (“Itaconate 10 mM”), added 10 min before the labeled substrate. 25 mM dithioerythritol (DTE) was added 1 min before the transport assay in a control (“Contr + DTE”) and an itaconate-treated aliquot (“Itaconate 10 mM + DTE”), respectively. The transport was stopped after 30 min by 1.5 mM NEM. The data are expressed as a percent of residual activity with respect to the control, without itaconate treatment, and are means ± SD from three independent experiments; they are significantly different from the respective control as estimated by Student’s *t*-test (** *p* < 0.01).

To investigate whether the interaction of itaconate with C136 was irreversible, the reducing agent dithioerythritol (DTE) was added to the reconstituted protein pretreated or not with the reagent. As shown in Figure 5, DTE was not able to reverse the inhibition of the transport activity of the carrier pre-incubated with 10 mM itaconate in agreement with the covalent and irreversible bond introduced by alkylation. The small activation exerted by the DTE addition was due to the action of the reducing agent on a fraction of the protein, which was oxidized by atmospheric oxygen [9] before reacting with itaconate, as evidenced by the activation of the untreated protein to a similar extent.

**Figure 6:**
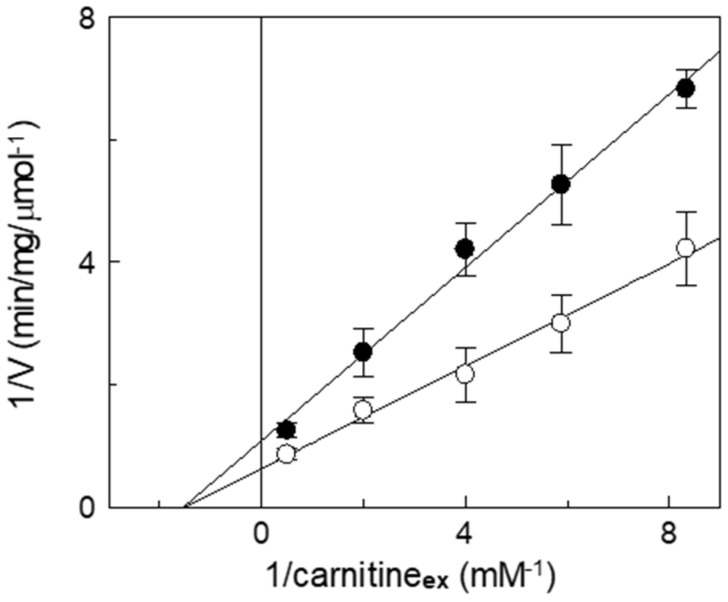
Kinetic analysis of the inhibition by itaconate of the recombinant CAC. The carnitine homo-exchange rate was measured, as described in Materials and Methods, adding [^3^H]carnitine at the indicated concentrations to proteoliposomes containing 15 mM internal carnitine in the absence (○) or in the presence of 10 mM itaconate, added 10 min before the labeled substrates (●). The transport was stopped after 10 min (i.e., within the initial linear range of the time course, see Materials and Methods) by 1.5 mM NEM. Experimental data are plotted according to Lineweaver–Burk as reciprocal transport rate vs. reciprocal carnitine concentrations. Values are means ± SD from three independent experiments.

Inhibition kinetics were also performed in the presence or absence of itaconate, and the data were used to draw a double reciprocal plot and to calculate the half-saturation constant (Ki). Figure 6 shows a non-competitive inhibition of WT, as indicated by the straight lines that intersect on the *X*-axis. The calculated Ki value for itaconate at pH 7.0 is 14 ± 2.6 mM. This pattern is typical of irreversible inhibition, again in agreement with the previously described data. Indeed, even though C136 is close to the substrate binding site [7,43], the irreversible inhibition leads to a non-competitive pattern. To confirm the reaction of itaconate with a target that is close to the substrate binding site, a protection analysis was performed. The reconstituted WT protein was incubated with 10 mM itaconate at pH 7.0, in the presence or absence of carnitine.

**Figure 7:**
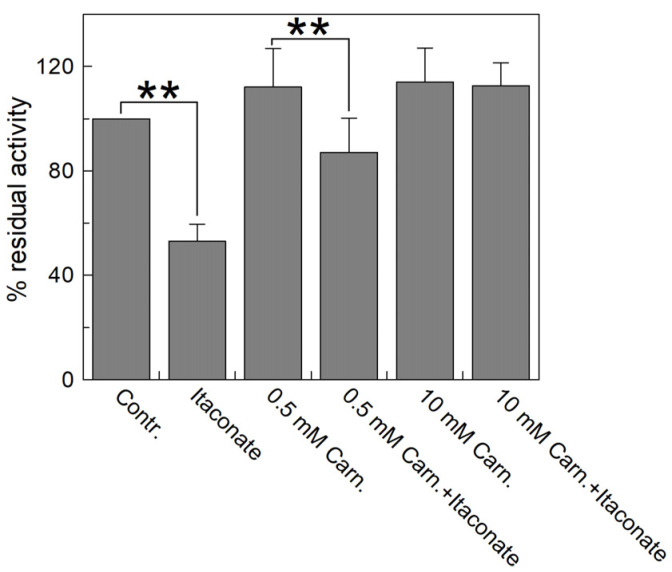
Substrate protection of WT CAC protein inhibition by itaconate. Carnitine at the indicated concentrations (0.5 and 10 mM) was added to proteoliposomes passed through a Sephadex G-75 column and incubated for 10 min with 10 mM itaconate. Then, the unreacted compound was removed by passing the proteoliposomes through a second Sephadex G-75 column. Transport was started by adding 0.1 mM [^3^H]carnitine to the proteoliposomes and stopped after 10 min with 1.5 mM NEM. Percent residual activity is reported with respect to the control without the addition of the inhibitor. The data were the means ± SD from three different experiments. They were significantly different from the respective control as estimated by Student’s *t*-test (** *p* < 0.01).

**Figure 8:**
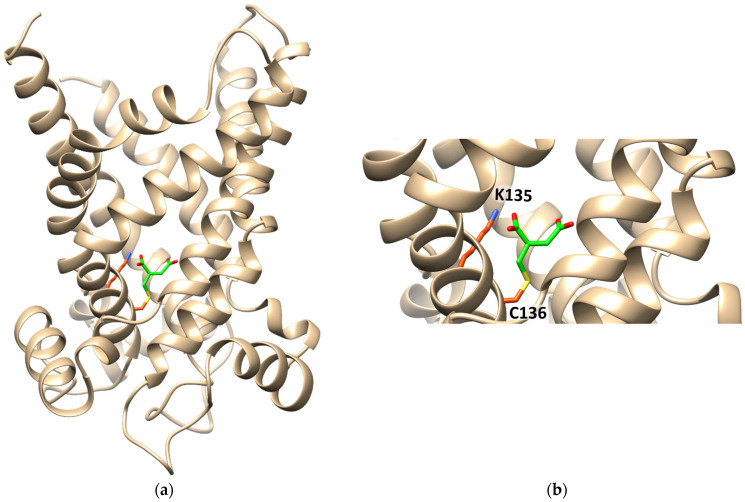
Ribbon diagram of the homology model of CAC, in the cytosolic open conformation, interacting with itaconate. (**a**) The protein is constituted by six transmembrane α-helices (depicted in tan) surrounding a central cavity in which C136 and K135 are located. Itaconate is highlighted in green. (**b**) Enlarged view of itaconte binding site. Covalent docking was performed with CovDock by Schrödinger. Docking visualization was performed with the UCSF Chimera v.1.14.

Figure 7 highlights that the inhibition observed with itaconate (about 55% compared to the control) decreased by preincubating the reconstituted transporter with carnitine. The substrate protection ranges from 80% to 100% in the presence of 0.5 and 10 mM carnitine, respectively, according to the location of C136 close to the substrate binding site (Figure 8) [44,45,46].

The interaction of itaconate with the CAC was also investigated by computational analysis (Figure 8). The data show the formation of a covalent bond between itaconate and C136. Interestingly, a Lys residue, namely K135, which is very close to Cys136, interacts with one of the carboxyl groups of itaconate by a hydrogen bond (Figure 8).

**Figure 9:**
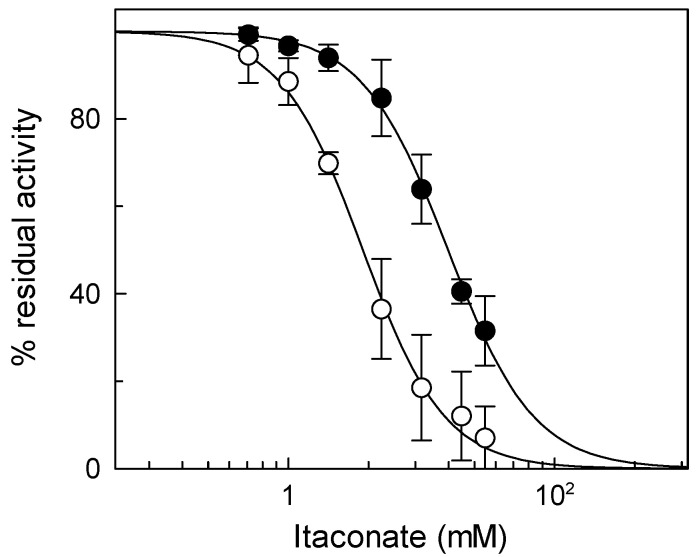
Dose–response analysis of inhibition by itaconate of WT protein (○) and the K135A (●) mutant was performed by measuring the transport activity with or without itaconate treatment. Transport activity (µmol/10 min/mg) is: WT 1.80 ± 0.31 and K135A 1.27 ± 0.32. The data are expressed as a percent of the own controls and are means ± SD from three independent experiments.

In a previous paper, we demonstrated that K135, a vicinal residue of C136, is responsible for the high sensitivity of the CAC to H_2_S [10]. To test whether the reactivity of itaconate for C136 is influenced by K135, too, a dose–response analysis of the metabolite on the WT and the mutant K135A was performed. Figure 9 shows that the IC_50_ value of the mutant (16 ± 2.6 mM) is about four times that of WT (3.8 ± 1.0 mM). This result confirms the crucial role of K135 in the reactivity of C136 toward itaconate (Figure 9).

### 3.5. Effect of Itaconate on the CAC in Cells

**Figure 10:**
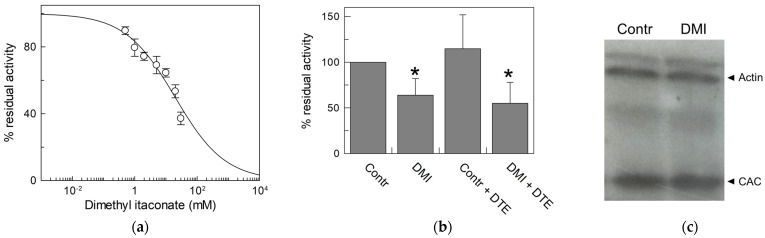
Effect of dimethyl itaconate (DMI) on CAC. (**a**) Dose–response analysis of inhibition by DMI of the recombinant CAC protein (○) was performed. Proteoliposomes were incubated with dimethyl itaconate at the indicated concentrations for 10 min. Thus, the transport activity was measured for 10 min by adding to proteoliposomes 0.1 mM [^3^H]carnitine. (**b**) Effect of DMI on CAC derived from HeLa cell lysate. CAC from mitochondria protein extract of HeLa cells treated with 10 mM DMI or vehicle (control) was reconstituted in proteoliposomes. Transport activity in the presence or absence of DTE was measured as described in Materials and Methods. Percent residual activity was reported with respect to the control. The data are the means ± SD from three different experiments, significantly different from the control (100%) as estimated by the Student’s *t*-test (* *p* < 0.05). (**c**) Western blot analysis using lysate from HeLa cells treated with DMI or vehicle (control) was performed to verify that the expression of CAC (32.5 kDa) did not change upon treatment with respect to actin (42 kDa) as the loading control. It is representative of three independent experiments.

To check the action of itaconate on the CAC in an intact cell system, dimethyl itaconate (DMI) was employed. It is the permeable uncharged analog of itaconate, used in intact cells to mimic the anti-inflammatory effect of endogenous itaconate [19]. Thus, we first tested the effect of DMI on the recombinant protein to ascertain its interaction with the CAC. Figure 10a displays the dose–response curve of the WT CAC incubated with dimethyl itaconate at pH 6.5. The calculated IC_50_ value is 20 ± 4.9 mM, indicating a lower affinity of the transporter for DMI than for the physiological compound itaconate; however, DMI is still suitable for testing its action in vivo. For this purpose, HeLa cells were treated for 45 min with 10 mM DMI or vehicle, and then mitochondria were isolated from cells (see Section 2). The CAC extracted from HeLa mitochondrial lysate was reconstituted and assayed for transport activity. Figure 10b shows that after in vivo administration of DMI [47], the CAC is strongly inhibited, indicating that endogenous itaconate can regulate the function of the CAC. The inhibition could not be reversed by DTE (Figure 10b), in agreement with the irreversible interaction with the protein in vivo.

## 4. Discussion

The data described in this work demonstrate that itaconate reacts irreversibly with C136 of the CAC, leading to the suppression of the transport function. Therefore, the compound represents the physiological counterpart of N-ethylmaleimide, a reagent that was identified as the most efficient alkylating compound of the CAC more than forty years ago. The observation that itaconate is a general Cys modifier in proteins [15,19] fits our findings; therefore, the CAC can be added to the list of itaconate targets. This novelty has a specific significance since the CAC inhibition may decrease the Fatty Acids Oxidation (FAO) rate. The itaconate post-translational modification can be assimilated to those previously described by other endogenous signaling molecules such as NO, GSSG, and H_2_S, further confirming that the CAC is a specific target for redox sensing and signaling. In this case, it may respond to the signaling of inflammation since itaconate is produced as a physiological response to inflammatory stimuli [48]. The possible inhibition of FAO could have a parallel and synergistic effect on the inhibition of SDH exerted by the same compound [48]. Interestingly, the molecular mechanisms of inhibition of SDH and the CAC are completely different. Inhibition of SDH shows a competitive mechanism [18], whereas inhibition of the CAC shows an irreversible non-competitive mechanism. In this respect, the irreversible inhibition of the CAC should guarantee a more persistent effect. Another important, albeit indirect, consequence of the irreversible inhibition of the CAC by itaconate may be a decrease in the production of the intermediate succinate in the TCA possibly acting synergistically with the inhibition of SDH by itaconate [3], further decreasing ROS production.

We have demonstrated in this work that the sensitivity to itaconate is a specific feature of the CAC. This is due to the peculiar microenvironment of C136 that has a greater propensity to give alkylation due to the vicinity of a basic residue, i.e., K135. Interestingly, also in KEAP1, one of the major players of itaconate action in inflammation, the target cysteine, i.e., C151, is flanked by a vicinal Lys residue, i.e., K150, as it emerges from the sequence analysis, exactly as it occurs in the CAC. This peculiarity renders the carrier sensitive to itaconate at a concentration close to the range reached by the compound (1.5–8 mM) when it is produced endogenously by overexpressed Irg1 [14]. Moreover, the in silico analysis of the itaconate–CAC interaction highlights that the metabolite, once bound to C136, interacts with K135 too (Figure 8), via an H-bond, indicating that the role of K135 may also be that of stabilizing the binding of itaconate in the center of the carrier hydrophilic cavity, where the substrate binds. Bound itaconate can then interfere with the substrate binding; indeed, the crucial residues for carnitine binding, i.e., K275, D179, and R178, are in the vicinity of C136 in the 3D structure of the hydrophilic cavity of the carrier [45]. This is also in line with the protection effect exerted by the substrate (see Figure 7) before the formation of the covalent bond. No effect can be expected by itaconate on the ORC or on the ANT that exhibits an effect of one or two orders of magnitude higher, respectively, than the endogenous concentration of the alkylating agent.

**Figure 11:**
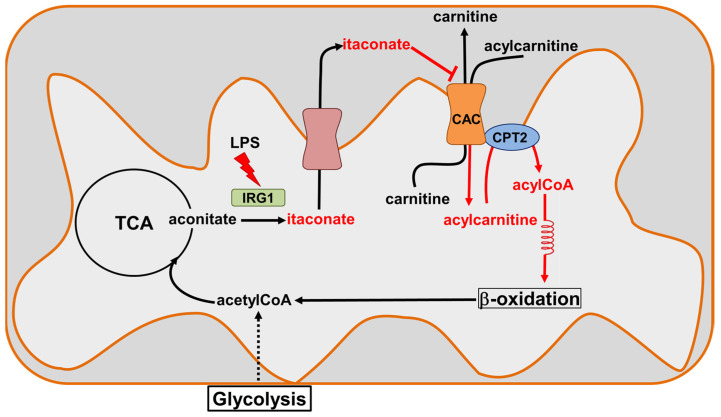
Sketch of CAC inhibition by itaconate. Itaconate is produced by Immune-Responsive Gene 1 IRG1(light green), the overexpression of which is induced during inflammatory states (lipopolysaccharide, LPS). Itaconate can cross the inner mitochondrial membrane through the dicarboxylic or tricarboxylic acid carriers (salmon) to reach the intermembrane space. The increase in itaconate in this compartment inactivates CAC (orange), inhibiting the entry of the acyl groups into the mitochondrial matrix and, hence, probably acting on the β-oxidation.

We hypothesize that the physiological significance of the inhibition of the CAC transport activity by itaconate is part of a complex system regulating the anti-inflammatory action of this metabolite [14]. When the cell is subjected to inflammatory stress, for example, in the case of macrophages activation by LPS (innate immunity), reactive oxygen species (mROS) are produced by mitochondria. In this context, in addition to the activation of a complex antioxidant mechanism useful to preserve the normal cell redox homeostasis [49], the overexpression of the enzyme Irg1 leads to the formation of itaconate, which the dicarboxylic or tricarboxylic acid carriers can transport to the intermembrane space (IMS) of the mitochondria [15]. In this compartment, favored by localized pH acidification [35], the reactivity of itaconate toward the CAC increases. This may have possible consequences on the mitochondrial FAO (Figure 11). We may also hypothesize that the interaction of the CAC with itaconate may represent an endogenous defense mechanism against the septic shock induced by bacterial infections, which is reported as a major cause of death, especially in elderly and immunosuppressed patients [50].

## 5. Conclusions

Briefly, in this paper, we described the inhibition of the CAC by itaconate, adding a novel molecular target to explain the mechanism of action of this pathophysiological compound.

## Data Availability

The datasets analyzed in this study are available from N.G. (first author) upon reasonable request.

## Supplementary Materials

The following supporting information can be downloaded at: https://www.mdpi.com/article/10.3390/biom13060993/s1, Figure S1: Effect of itaconate on the native mitochondrial adenine nucleotide transporter (ANT).
