# Supplementary material for: Inhibition of the Mitochondrial Carnitine/Acylcarnitine Carrier by Itaconate through Irreversible Binding to Cysteine 136: Possible Pathophysiological Implications
Source: Biomolecules. 2023 Jun 15;13(6):993. doi: 10.3390/biom13060993 (PMC10296061; doi:10.3390/biom13060993)
Supplement: Supplementary file 1 [file biomolecules-13-00993-s001.zip › biomolecules-2423117-supplementary.pdf]

**Supplementary Figure**

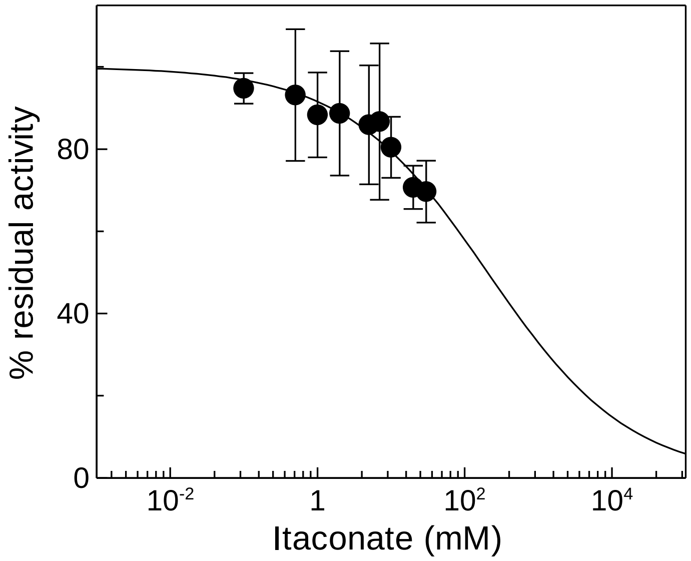


**Figure S1.** Effect of itaconate on the native mitochondrial adenine nucleotide transporter (ANT). Dose-response analysis for itaconate inhibition was carried out using proteoliposomes reconstituted with native ANT. After 10 min of itaconate incubation, transport activity was started by adding 0.1 mM [$^3$H]carnitine and stopped after 10 min as described in Materials and Methods. Percent of residual activity with respect to the control, without itaconate treatment, was reported. The values are the means ± SD from three independent experiments.
